# Involving young people in cyberbullying research: The implementation and evaluation of a rights‐based approach

**DOI:** 10.1111/hex.12830

**Published:** 2018-10-10

**Authors:** Rebecca Dennehy, Mary Cronin, Ella Arensman

**Affiliations:** ^1^ School of Public Health University College Cork Cork Ireland; ^2^ National Suicide Research Foundation University College Cork Cork Ireland

**Keywords:** children's rights, collaboration, cyberbullying, patient and public involvement, qualitative research, young people

## Abstract

**Background:**

Cyberbullying is an international Public Health concern. Efforts to understand and address it can be enhanced by involving young people. This paper describes a rights‐based collaboration with young people in a qualitative exploration of cyberbullying. It describes the establishment, implementation and evaluation of a Young Person's Advisory Group as well as identifying the impact on the research process and the young people involved.

**Methods:**

Sixteen postprimary school students met with researchers on five occasions in a youth centre. Sessions focused on building the young people's capacity to engage with the research, designing the qualitative study, interpreting study findings and evaluating the collaboration process.

**Results:**

The Advisory Group highlighted a lack of understanding and appropriate action with regard to cyberbullying but believed that their involvement would ultimately help adults to understand their perspective. Evaluation findings indicate that members were supported to form as well as express their views on the design, conduct and interpretation of the research and that these views were acted upon by adult researchers. Their involvement helped to ensure that the research was relevant and reflective of the experiences, interests, values and norms of young people.

**Conclusion:**

Young people can contribute a unique perspective to the research process that is otherwise not accessible to adult researchers. The approach described in this study is a feasible and effective way of operationalizing young people's involvement in health research and could be adapted to explore other topics of relevance to young people.

## 1 BACKGROUND

Cyberbullying is an international public health concern and is a serious problem facing young people today.^1^, ^2^ There is a lack of consensus regarding conceptual and operational definitions of cyberbullying; however, in an attempt to unify definitional inconsistencies in the literature, it has been defined as “…*behaviour performed through electronic or digital media by individuals or groups that repeatedly communicates hostile or aggressive messages intended to inflict harm or discomfort on others*.”^3^ It is estimated that 10%‐40% of children and young people have experienced cybervictimization.^4^ Cyberbullying has a detrimental effect on the psychological, physical and social well‐being of both victims and perpetrators.^4^, ^5^, ^6^ It is associated with anxiety and suicidal behaviour (fatal and nonfatal) and has a stronger relationship with depressive symptoms and suicidal ideation than traditional bullying.^4^, ^5^, ^7^ Despite the negative impact on the health of young people, evidence‐based prevention and intervention strategies are lacking.^8^, ^9^ Cyberbullying is a contemporary problem facilitated in recent years by a rapid growth in information and communication technology. Adults do not have first‐hand experience of being immersed in social media in their youth;^10^ therefore, the development of effective interventions requires a thorough understanding of cyberbullying^11^, ^12^ from the perspective of young people.^1^, ^13^ Existing research is predominantly quantitative in nature, and young people's voice is largely absent from the current discourse.^14^, ^15^, ^16^ The omission of young people's perspective may lead to a misinterpretation of their needs and misguided prevention and intervention strategies.^13^ It has been suggested that collaborating with young people as coresearchers could enhance efforts to understand and address cyberbullying.^10^, ^15^, ^16^

Patient and public involvement in research is increasingly expected to be an inherent part of research development. It is defined as “*research being carried out ‘with’ or ‘by’ members of the public rather than ‘to’, ‘about’ or ‘for’ them*” and refers to the active involvement of patients/public in research “*rather than the use of people as participants in research or as research subjects.”* 17 It is founded on the principle that people have a right to express their views on matters that affect their lives^18^ and it has been shown to enhance the quality, appropriateness and relevance of health research.^19^ Involvement encompasses collaboration, which refers to an on‐going partnership between researchers and patients/public and shared decision making.^20^ This approach is thought to be more effective than once off consultations or sporadic involvement in the research process.^19^ As enshrined in Article 12 of the United Nations Convention on the Rights of the Child (UNCRC), it is the right of young people to have a say in matters that affect them.^21^ Collaboration with young people has the potential to increase the relevance of research, enhance methodological rigour, provide rich data on cyberbullying and positively impact on the young people involved.^1^, ^20^, ^22^, ^23^, ^24^, ^25^ The way that research is conducted and the methods that are used to access young people's views can impact on those who are involved as research participants and ultimately on health outcomes.^26^ As competent social actors and “digital natives,”^27^ young people, in the role of coresearchers, can provide a unique perspective on the design, conduct and interpretation of cyberbullying research to facilitate the appropriate and meaningful participation of their peers as research participants.^10^

Published examples of collaborations with young people in health research are limited,^20^, ^28^ particularly in regard to cyberbullying research.^15^, ^29^, ^30^ Additionally, it has been noted that young people are rarely asked about their involvement in research,^24^, ^31^, ^32^ and therefore, insight into young people's views on methods and approaches to collaboration are lacking. This study presents a rights‐based approach to collaborating with young people in a qualitative exploration of cyberbullying. It describes the establishment, implementation and evaluation of a Young Person's Advisory Group as well as identifying the impact on the research process and the young people involved. Young people's involvement in the study is reported in line with guidance for reporting patient and public involvement in research (GRIPP2).^33^

## 2 METHODS

### 2.1 Rights‐based approach

The study was informed by Lundy's Model of Participation,^26^, ^32^, ^34^ which conceptualizes Article 12 of the UNCRC.^21^ This model identifies four key chronological concepts underpinning the effective realization of young people's participation: (a) *space*—children must be given the opportunity to express a view in a space that is safe and inclusive, (b) *voice—*children must be facilitated to express their views, (c) *audience*—the view must be listened to and (d) *influence*—the view must be acted upon as appropriate.^26^, ^34^ Lundy's Model highlights that Article 12 does not exist in isolation and should be recognized in line with other children's rights including the right to guidance from adults (Article 5) and the right to seek and impart appropriate information (Article 13 and 17).^32^, ^34^ A rights‐based approach to collaborating with young people therefore requires that young people are supported in not only expressing their views but also in forming them.^32^

### 2.2 Adult researchers

The adult researchers have experience of working with young people in school and youth work settings, in community and mental health research and in participatory and qualitative research methods.

### 2.3 Recruitment of schools

The recruitment of schools commenced in spring 2016 with a view to beginning work at the start of the 2016/2017 school year. Four schools in a large town in the Republic of Ireland were invited to participate. These included an all‐girls voluntary secondary school (non‐fee‐paying), an all‐boys voluntary secondary school (non‐fee‐paying), a coeducational private school (fee‐paying) with a mix of day students and boarders and a coeducational vocational school (non‐fee‐paying) in receipt of additional supports to address educational disadvantage and social exclusion.^35^ An information sheet was sent to the principal of each school and during follow‐up meetings all four schools agreed to participate. Written consent was obtained to formalize the agreement. A contact person was elected by each principal to act as a link between the adult research team and the school.^36^, ^37^

### 2.4 Establishment of the Advisory Group

Transition Year is an optional 1‐year programme in the fourth‐year of postprimary education in Ireland. Taken after the Junior Certificate (1st‐3rd year) and before the Leaving Certificate (5th and 6th year), Transition Year promotes the personal, social, vocational and educational development of students without the pressure of state examinations.^38^ These students were therefore considered well‐placed to be involved in the Advisory Group. In September 2016, the lead researcher spoke to Transition Year students about the project and distributed information sheets. Students were advised that their membership of the Advisory Group would be known to others. Transition Year Coordinators in each school elected four students from those interested to sit on the Advisory Group. Ten female and six male students participated, all were 16 years old. Written consent was obtained from both young people and a parent/guardian and forms were returned to the school.^37^, ^39^

### 2.5 Ethical considerations

Ethical approval for the study was granted by the University Clinical Research Ethics Committee. The study was conducted in line with ethical^40^, ^41^ and child protection guidelines.^42^, ^43^, ^44^ It was agreed with schools that any concerns about the safety of a young person during the study would be addressed in line with their school's child protection policy and standard operating procedure.^37^ The Guidance Counsellor in each school was available as a support, as was the local Youth Service. The lead researcher's contact details and relevant helplines were also provided.

### 2.6 Procedure

**Figure 1:**
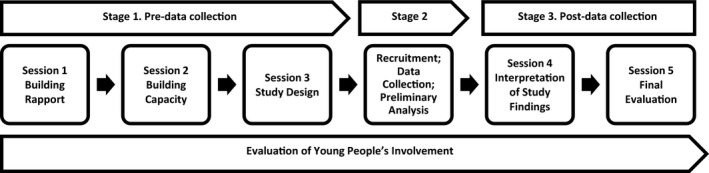
Process of collaborating with Young Person’s Advisory Group in qualitative research

The Advisory Group met with adult researchers for 5 two‐hour research sessions in the 2016/2017 school year. These were held in a youth centre and were attended by a Youth Worker and two adult Research Officers. A kitchenette was available to prepare snacks, which were provided at each session. The work was conducted in three stages (Figure 1). Sessions focused on building the young people's capacity to engage with the research process and the issues surrounding cyberbullying, designing a qualitative study, interpreting the findings of the study and evaluating young people's involvement in the Advisory Group; the latter was informed by guidelines for evaluating participation work with young people.^45^, ^46^

#### 2.6.1 Stage 1: Session 1—building rapport

Session 1 focused on building rapport among the research team. Icebreakers were used to ease inhibitions, build trust and create an open atmosphere.^47^ In an attempt to alleviate any concerns and manage expectations, the Advisory Group were invited to write their “hopes and fears” for their involvement on sticky notes, which were then discussed. “Hopes” can reveal motivations for participation; therefore, this information also contributed to the on‐going evaluation process.^45^, ^48^ Terms of Reference for the Advisory Group were reviewed collaboratively and approved. As is good practice in group facilitation,^49^ and in working with young people, a group contract was developed to set out the fundamental rules of the group (Table 1).^49^, ^50^ The Advisory Group were reassured that discussion would be confidential and anonymized except in the event of a disclosure of potential risk to a young person or to others.^31^, ^39^ They were reminded on an on‐going basis that they were free to withdraw from an activity or from the process as a whole at any time.^41^, ^51^, ^52^ They selected “*#SocialSesh”* as the name for the Advisory Group as they felt it represented their interest in social media and social research, and demonstrated the social aspect of the group.

**Table 1:** Terms of Reference and group contract for the Young Person's Advisory Group

**Terms of Reference**
Work with adult researchers, youth worker and other advisory group members as part of a team
Contribute a young person's point of view
Advise on the best ways to talk to postprimary school students about cyberbullying
Comment on the research findings
Identify key issues to be addressed to help those affected by cyberbullying
**Group Contract**
No mobile phones
No bullying
Participate
Maintain confidentiality where appropriate
Listen to and respect group members
Have fun

#### 2.6.2 Session 2—building capacity

Session 2 focused on building the Advisory Group's capacity to engage with the research and the issues surrounding cyberbullying.^32^, ^53^ It aimed to enable the Advisory Group to express their existing views or form new ones based on the interaction with the information generated, their peers and the adult researchers.^32^ Brief training in Public Health research was delivered to enable them to make informed contributions to the study design.^19^ Key topics included “What is public health?”; “What is research?”; “The cycle of a research project”; “Research methods”; and “Research ethics.”

Strategies to enable the Advisory Group to reflect on and discuss cyberbullying were informed by the literature on capacity building and participatory methods.^32^, ^53^, ^54^ A topic guide^55^ developed at the University of Toronto to explore cyberbullying with young people was used to inform discussion topics, which included defining cyberbullying, cyberbullying behaviours, motivations, consequences and coping, and reporting. Participatory enabling techniques were implemented to stimulate thinking and to facilitate the Advisory Group to refine and express their views.^56^ These techniques provided further insight into the nature of the research and allowed for adaptation of the topic guide for use later in the project. The nature of cyberbullying and its relationship to traditional bullying was discussed. Walking debates, a tool to encourage discussion and the formation of views,^57^, ^58^ were conducted to enable reflection on the role of gender and setting in cyberbullying, to identify the characteristics of those impacted by victimization and perpetration and to explore current prevention and intervention efforts. “Flexible Brainstorming”^59^ and “Sorting and Ranking”^48^, ^59^ facilitated discussion about the media through which cyberbullying takes place, and the carousel technique^48^ was used to enable the Advisory Group to consider motivations for cyberbullying and the impact on those involved (see Appendix S1 for detailed description). At the end of the second session, the Advisory Group wrote their thoughts about the day on sticky notes as part of the on‐going evaluation.

#### 2.6.3 Session 3—study design

In the third session, the Advisory Group advised on the recruitment of study participants and data collection tools and strategies. At the end of Session 2, each member of the Advisory Group was given a draft copy of an information sheet and a consent form to review at home. They brought these to the third session where they presented their feedback on the accessibility of the content before approving the documents for use.

The Advisory Group suggested that the sample should include second‐ (aged 14), fourth‐ (aged 16) and fifth‐year students (aged 16‐17). They recommended excluding those preparing for state examinations (3rd and 6th year) as they would have constraints on their time and also first‐year students. They felt that as first‐year students were new to the school and still “*getting used to their environment,*” they might be intimidated by the process or would not take the process seriously. One member stated: “*I feel if you ask a first year any of that he wouldn't take it seriously, like he wouldn't get the seriousness of it.”* The Advisory Group decided that they would like to be involved in the recruitment process suggesting that they would be better able than adult researchers to encourage the participation of their peers.

The Advisory Group debated the merits of various approaches to collecting qualitative data from the students in their schools. They suggested that focus groups would be less *“intimidating”* for students than one‐to‐one interviews. They stressed that school staff should not be in attendance at the focus groups as they felt it would compromise the openness of the conversation with one member highlighting: “*you wouldn't feel like you could be completely honest, it would have to be with like people who are not in the school*.” It was agreed that the participants in each focus group should be from the same year group to promote comfortable discussion. The Advisory Group recommended that icebreakers and group contract development should be included at the beginning of each focus group.

Having developed an understanding of cyberbullying and related issues during the capacity building session, the Advisory Group reviewed the topic guide and adapted it for use with participants in the Irish postprimary school setting. As the topic guide was originally used in one‐to‐one interviews, the questions were rephrased to suit a focus group setting. To ensure confidentiality and encourage openness, it was decided that participants would not be asked directly about their personal experiences. Prompts related to the taking and distribution of “*nude images*” through social media were added to the topic guide as the Advisory Group viewed this as a key issue for Irish young people.

The final task with regard to study design was to agree a protocol for the provision of support to any participant experiencing distress. Initially, the Advisory Group wanted to make themselves available in their respective schools. However, the adult researchers believed that this may deter participants from seeking support, put a vulnerable participant at risk or create an unnecessary burden for Advisory Group members. With reference to Article 19 (right to be safe) and Article 3 (best interests of the child) of the UNCRC, it is the responsibility of adult researchers to ensure the safety of the young people involved in the research and to make decisions in their best interests.^21^ Therefore, given the association between cyberbullying and suicidal behaviour and the potential risk of harm to the young people involved, the adult research team decided that participants seeking support would be directed to the lead researcher, their Guidance Counsellor or the Youth Worker involved in the study. Relevant helplines would also be provided. The reasoning for the decision was discussed openly with the Advisory Group, and they accepted the rationale. At the end of the session, as is custom on a popular social media platform, the Advisory Group were invited to write their thoughts about the day in 140 characters or less. This concise feedback contributed to the on‐going evaluation.

#### 2.6.4 Stage 2: recruitment and data collection

The next stage involved recruitment to the focus groups. The lead researcher visited individual second‐, fourth‐ and fifth‐year classes with Advisory Group members in their respective schools. Members explained the nature and purpose of the study and encouraged their peers to participate. Interested students were provided with an information sheet and asked to return completed consent forms, in an envelope provided, to the school contact person. These were collected by the lead researcher. The Advisory Group therefore were not aware of the identity of the participants. In total, 64 students (30 male and 34 female, aged 14‐17) agreed to participate and subsequently 11 focus groups were conducted across the four schools using the format codesigned with the Advisory Group.

#### 2.6.5 Stage 3: Session 4—interpretation of findings

Audio from the 11 focus groups was transcribed, and a qualitative analysis was conducted by adult researchers. Consensus was reached on the identified themes, and preliminary findings were presented to the Advisory Group during Session 4. They were asked whether they believed the research findings to be reflective of young people's experience of cyberbullying and to identify what they perceived to be the key issues within the findings. The carousel technique^48^ was used to facilitate the Advisory Group in considering what needs to change to address cyberbullying and how this change can be achieved.

#### 2.6.6 Session 5—final evaluation

In Session 5, the final evaluation of the Advisory Group's involvement in the research process was conducted. Participatory techniques generated qualitative data, which were coanalysed with the Advisory Group using the principles of thematic analysis.^60^ Discussion topics included motivations for involvement, the role and impact of the Advisory Group, the suitability of the approach and the impact on the young people involved. A framework approach^61^ was later applied by adult researchers to structure the findings and to establish if the elements of Lundy's Model of Participation^34^ were present. This enabled the exploration of a priori objectives but allowed themes to be identified through the Advisory Group's interpretation of the data. Handwritten data, photographs, interpretations and summaries produced throughout the sessions were recorded electronically along with notes taken by adult researchers. NVivo 11 was used to manage the data. Open‐coding was conducted, and codes were grouped according to identified themes. Themes were mapped onto a framework informed by Lundy's Model of Participation,^34^ which outlines the four elements necessary for meaningful participation in accordance with Article 12 of the UNCRC: Space, Voice, Audience and Influence.^21^ Findings were sent via email to the Advisory Group for “member‐checking.”^62^ Five members responded; all five were in agreement with the findings, and no changes were suggested. At this point, Advisory Group members had completed Transition Year and had commenced preparation for their state examinations; therefore, researchers did not follow up with those who chose not to engage.

### 2.7 Recognition of involvement

Advisory Group members were awarded personalized Certificates of Participation. Additionally, members requested and were provided with help to formulate details of their new experience, training, and skills for inclusion in their curricula vitae and Transition Year Portfolios.^38^

## 3 EVALUATION FINDINGS

All 16 members of the Advisory Group remained involved for the duration of the process; on only one occasion was a member absent due to a conflicting commitment. The Advisory Group's input is summarized in Table 2; findings from the evaluation of their involvement are presented with supporting quotes in Table 3.

**Table 2:** Input of Young Person's Advisory Group to research process

**Young Person's Advisory Group**
Development of Terms of Reference and Group Contract for Young Person's Advisory Group
Selection of name for Young Person's Advisory Group, that is #SocialSesh
**Study design**
Identification of key issues of relevance to Irish young people with regard to cyberbullying
Development of study materials, that is information sheet, consent form, helpline information
Development of data collection tools and strategies, for example use of focus groups to collect data, use of icebreakers and group contract at the beginning of focus group sessions
Adaptation of topic guide for use in focus groups with students in postprimary schools
Selection of study sample, that is second‐, fourth‐ and fifth‐year postprimary school students
Development of recruitment strategy
**Study conduct**
Recruitment of peers to participate in focus groups
**Interpretation of study findings**
Interpretation of findings from focus groups with postprimary school students
Identification of key issues for consideration in intervention development
**Evaluation**
Co‐analysis of data collected during final evaluation of Young Person's Advisory Group
Review of evaluation findings

**Table 3:** Young person's Advisory Group's evaluation of involvement in the research

Themes	Quotations to illustrate young people's experience
**Motivations**	
Relevance of the research topic	“the research is relevant to people my age” “we are going through it and it is something that concerns us”
Fear of cybervictimization	“getting hurtful comments [online]” “being judged [online]” “being afraid in your own home” “everyone laughing at you online” “It [cyberbullying] is with you 24/7”
Lack of understanding from parents and schools	“people [adults] think it's [cyberbullying] something different” “the difference between what adults and young people think [about cyberbullying], that is a problem” “I think sometimes with your parents they might find it hard to understand what you are going through because they didn't have phones or anything they had like…bicycles” “help you understand what it [cyberbullying] means to us”
Lack of appropriate action from schools	“They [school] just don't care much…they care more about the school's reputation than the actual mental wellbeing of their students” “They are just so out of touch with everyone like. The cyberbullying campaign was like a cartoon of someone sending like a text on a Samsung like “I hate you” sad face. Like that just doesn't happen” “It is not like anything that actually happens, it is not realistic and you can't relate to it” “They were like how many people have their Facebook private and then like the hands went up and they didn't count them like, they didn't say why you should have your account on private or anything like that” “Like when we had a talk it was kind of just like OK now tell everyone you have had your cyber‐talk” “to find ways to prevent cyberbullying instead of ignoring it”
Altruism	“make people more aware of cyberbullying” “to help people cope and deal with cyberbullying” “to help bullies understand the impact of their actions”
Learning opportunity	**“**to understand the impact cyberbullying has on people” “to get a better knowledge of cyberbullying and cyberbullies” “to share my view on cyberbullying and see if other people have the same view”
Cynicism	“not helping at all with the project”
**Space**	
Physical environment	“comfy couches around and stuff and bean bags” “nice and cosy”
Social environment	“a good experience to talk about things that we hadn't talked about in as much detail before” “an important topic that we could be open about” “it was easy to put forward ideas” “you do not have any previous opinion of who we are so we can just be completely open and honest and that is how you see us”
**Voice**	
Understanding of the issues being researched	“I feel that I have a better understanding of cyberbullying, better on a whole new level” “The focus group helped to give an insight into cyberbullying”
Peer interaction	“I found it interesting to share and see others views”
**Audience**	
Feeling listened to	“everyone is listened to” “we were listened to”
Feeling valued	“they [adult researchers] greatly appreciate your thoughts and opinions”
Recognition of young people's perspective	“We told you how it [cyberbullying] happens” “you [adult researcher] kind of know how we feel, how it [cyberbullying] works, a lot of older people wouldn't”
**Influence**	
Views acted upon	“you designed it [the study] around what we were saying.” “I think it [young people's involvement] made the results more accurate than if only an adult were to do it”
Making a difference	“I feel like I have really changed something” “Really good way to make a difference”
**Personal impact on the Young Person's Advisory Group**	
Positive experience	“Memorable” “Really good fun experience” “It was lit fam” “Made new friends and had loads of fun” “I really enjoyed contributing”
Knowledge and understanding	“I understand how not to take cyberbullying personally as I know the reasons behind it” “Taught me ways to help” “I told loads of people what I learned” “Amazing information learned”
Personal development	“Increased confidence” “Getting out of my comfort zone” “Good to try different things” “I can't wait for what will come next”

### 3.1 Motivations for involvement

The Advisory Group were motivated primarily by the relevance of the research topic to their lives. They highlighted that cyberbullying was an on‐going concern and that many live in fear of cybervictimization. Members believed that there was a lack of understanding and appropriate action from parents and schools with regard to cyberbullying and that this was affecting efforts taken to address it. They highlighted that they could not relate to the content of existing cyberbullying interventions but believed that through their involvement they would help adult researchers understand the reality of the situation faced by young people and incite relevant action. Altruism was a key motivating factor. They articulated the hope that through their involvement they would raise awareness of cyberbullying and help both victims and perpetrators. While all of the members were enthusiastic about their involvement, some were cynical about the value of their contribution, unsure about how they could help with the project and concerned that their views might not be taken seriously.

### 3.2 Space

Efforts to create a safe and appropriate physical and social space appear to have been successful. The Advisory Group reported that they felt comfortable in the youth centre. They reported that they were facilitated to express their views on cyberbullying, stating that it was easy to put forward ideas because of a nonjudgemental space and an encouraging environment that fostered open discussion. They valued the opportunity for involvement and the space to discuss a topic that was of interest and relevance to young people.

### 3.3 Voice

Findings indicate that Advisory Group members were supported to form, as well as express, their views. While research training was provided during the capacity building session, it was the knowledge generated through the interaction with their peers and adult researchers that they valued more in supporting their involvement in the design, conduct and interpretation of the research. They highlighted that this had given them a deeper understanding of the issues under research.

### 3.4 Audience

The Advisory Group reported that they were listened to by their peers and adult researchers throughout the process. They perceived that their thoughts and opinions were valued and appreciated and that their position on cyberbullying and related issues had been recognized by the adult researchers. They highlighted that this was not normally their experience when interacting with adults about the issues facing young people.

### 3.5 Influence

The Advisory Group members reported that their views had been acted on during the course of the research. They believed that they had contributed directly to the study design and that the decisions they made were implemented in the conduct of the research. They claimed their involvement as coresearchers had improved the research process and made the findings of the qualitative study more accurate than if only adults were involved in the research. A sense of achievement was described based on a belief that they had made a difference to the study but also in being a voice for young people and ultimately in helping those affected by cyberbullying.

### 3.6 Personal Impact on Advisory Group members

All members described a positive social and learning experience during which they made new friends and had fun. They highlighted increased knowledge and understanding with regard to cyberbullying. Many members applied this learning to their own lives articulating that they now felt more equipped to cope with cyberbullying and to help others affected by it. They reported that they felt more confident because of their involvement and described satisfaction in stepping out of their comfort zone and trying something new.

### 3.7 Recommendations of the Advisory Group

As they attended the sessions during school hours, the Advisory Group were required to wear their school uniforms. They suggested that it would have been preferable to wear their own clothes as this made it easier for them to express themselves. They recommended that an additional session between Session 3 (Study Design) and Session 4 (Interpretation of Findings) would be useful as they found the time gap of 5 months too long. They suggested that the added session could provide an update on recruitment and data collection. Members felt that the rights‐based, participatory approach was successful and suggested *“expanding the topics of conversation”* to explore other areas of relevance to young people.

## 4 DISCUSSION

This study presents a rights‐based approach^26^, ^32^, ^34^ to collaborating with young people as coresearchers in a qualitative study of cyberbullying. It contributes a worked example to the limited body of knowledge on collaborating with young people in cyberbullying research^15^, ^16^, ^29^, ^30^ and in health research more broadly.^20^, ^28^ It reports a systematic evaluation of young people's involvement in the research process, an area which has been neglected in previous studies.^31^, ^32^, ^63^ Findings suggest that collaboration with young people is feasible and beneficial to the research process and those involved.

Echoing findings from previous research,^64^ at the outset of the process, some Advisory Group members expressed cynicism about the value of their contribution. This is likely as a result of experiencing tokenistic participation in which young people are apparently given a voice but in fact have little or no choice within the space provided or opportunity to formulate their own opinions.^13^, ^65^ Findings indicate that the elements necessary for the effective realization of young people's participation were present in this study.^13^, ^34^ The implementation of a rights‐based framework^26^, ^34^ strengthened young people's involvement and assured their right to have a say on an issue that affects them.^21^ Supporting the Advisory Group to form as well as express their views on cyberbullying ensured that their involvement, and the involvement of their peers as research participants, was meaningful.^32^ The study was adult‐initiated and involved shared decision making with the Advisory Group, placing it at Level Six of Hart's Ladder of Young People's Participation.^65^ Given the association between cyberbullying and suicidal behaviour and the potential risk to the young people involved in the Advisory Group, and as research participants,^4^, ^5^, ^6^, ^7^ this was found to be an appropriate level of participation. In keeping with a rights‐based approach,^34^ shared decision making enabled adult researchers to give due weight to the views of the Advisory Group but also to make decisions, when necessary, in their best interests (Article 3) and to ensure their safety (Article 19).^21^, ^34^

Motivations for participating in the Advisory Group were similar to those reported in a previous account of young people's participation.^31^ Members were motivated primarily by the relevance of the research topic to their lives. Effective intervention development requires a thorough understanding of the behaviours associated with cyberbullying^11^, ^12^ from the perspective of young people.^1^, ^13^ However, the advisory group highlighted a lack of understanding and appropriate action from parents and schools. Concurring with previous research,^13^ findings suggest that the omission of young people's voice in efforts to understand and address cyberbullying has led to a misinterpretation of their needs and misguided prevention and intervention strategies, particularly in the school setting. The present study underlines the importance of involving young people in efforts to understand and address cyberbullying.^16^ It is reportedly difficult to maintain young people's involvement in research;^23^, ^66^ however, all 16 members of the Advisory Group remained involved for the duration of the process and reported a fun and memorable experience. It is likely that their on‐going involvement was enabled by the nature of Transition Year and the conduct of sessions during school hours. Findings from this study indicate that the use of participatory enabling techniques contributed to open and honest discussion and to the positive experience reported by the Advisory Group. This supports previous research which suggests that young people enjoy activity‐oriented methods and that these can facilitate the discussion of difficult topics.^31^, ^67^ The collaboration was also an enjoyable and beneficial process for the adult researchers. The knowledge coconstructed during the capacity building session enabled adult researchers to approach data collection and analysis in a more informed manner. The Advisory Group's involvement in the interpretation of study findings, an area which is often neglected in efforts to involve young people in research,^68^, ^69^ revealed a unique perspective on the issues to be considered in the development of cyberbullying interventions.

The local youth service provided a safe, appropriate^26^ and youth‐friendly space for the Advisory Group sessions at no cost to the project. Monetary costs associated with the process were low and related to the purchase of refreshments and materials. Due to a limited budget, it was not possible to pay members for their time; however, there was no expense involved for the Advisory Group. Similar to an Advisory Group in another Irish study,^28^ members requested help in updating their CV's to reflect their new skills and experience, suggesting that this is a valued practice for young people. As in previous accounts of patient and public involvement,^19^ the practical aspects of involving young people were time‐consuming with the process described in this study taking 15 months from inception. The initial recruitment of schools to the project was a challenge; however, commencing recruitment in the school year prior to the school year when the study began^36^ proved beneficial as it allowed researchers adequate time to negotiate access with gatekeepers without impacting on the time spent working with the Advisory Group. The option to appoint a contact person^36^ for the study was welcomed by principals as it assured them that their workload would not be increased, thereby facilitating their participation.

### 4.1 Strengths and limitations

The implementation of a rights‐based model to frame young people's involvement^21^, ^26^, ^32^, ^34^ strengthened this study, and the experience and skills of the adult research team contributed to its safe and effective conduct. Recruiting through schools is more likely to result in a representative sample than recruiting via youth services or other channels. However, young people's behaviour in schools is influenced by the expectations and norms of that environment, which may encourage them to contribute perspectives considered socially desirable in that context.^31^ Holding the Advisory Group sessions in the youth centre facilitated the meeting of students from four different schools and enabled members to express their views freely. While focus groups were held in schools, the involvement of the Advisory Group in designing the study helped to create a safe and appropriate space within this setting, allowing for the meaningful participation of their peers as research participants. The Advisory Group evaluation was conducted with the adult researchers involved throughout the project, and this may have influenced responses. However, the strong rapport built over the course of the collaboration and the use of participatory methods in the evaluation, which anonymized the personal contributions of the members, may have contributed to more honest feedback.

## 5 CONCLUSION

Young people can provide a unique perspective on the design, conduct and interpretation of research that is otherwise not accessible to adult researchers. Collaboration can help to ensure that the research process and resultant outputs are reflective of the experiences, interests, values and norms of young people, thereby increasing the relevance and appropriateness of intervention and policy development. The approach described in this paper enabled the meaningful participation of young people as coresearchers and as research participants. It is a feasible and worthwhile way of operationalizing young people's involvement in health research and could be adapted to explore other topics of relevance to young people. It is intended that the findings from the on‐going qualitative study conducted with the Advisory Group will inform the development of relevant and appropriate interventions to tackle cyberbullying in young people.

## COMPETING INTERESTS

None to declare.
