# Supplementary material for: Involving young people in cyberbullying research: The implementation and evaluation of a rights‐based approach
Source: Health Expect. 2018 Oct 10;22(1):54–64. doi: 10.1111/hex.12830 (PMC6351412; doi:10.1111/hex.12830)
Supplement: Supplementary file 1 [file HEX-22-54-s001.docx]

**Description of Participatory Enabling Techniques**

**Walking Debates**

Walking debates, a tool to encourage discussion and the formation of views,^1,2^ were conducted to enable reflection on the role of gender and setting in cyberbullying, to identify the characteristics of those impacted by victimisation and perpetration and to explore current prevention and intervention efforts. Two signs with the words “*I agree*” and “*I disagree*” were placed on the wall on either side of the room. Statements such as “*Girls are more likely to be victims of cyberbullying than boys”* were read aloud. Advisory Group members were invited to walk to the sign that best reflected their view or stand in the middle if they were unsure. To encourage dialogue, they were encouraged to defend their position and to move if their view changed over the course of the debate.

**Flexible Brainstorming and Sorting and Ranking**

‘Flexible Brainstorming’ ^3^ and ‘Sorting and Ranking’ ^3,4^ facilitated discussion about the media through which cyberbullying takes place. The YPAG were provided with flipchart paper, sticky notes, card, and markers and invited to use the materials to depict the media through which cyberbullying takes place using one sticky note or piece of card per idea. They were then asked to sort their ideas into meaningful groupings. Through discussion and a process of retaining or removing certain items a list of the media they believed to facilitate cyberbullying was formed and items were ranked according to the perceived risk of victimisation. This was then used as a tool to enable reflection and discussion about the nature of cyberbullying in different outlets.

**The Carousel Technique**

The Carousel technique^4^ was used to enable the YPAG to consider motivations for cyberbullying and the impact on those involved. Four tables were set up with markers and a sheet of flipchart paper with one question on it such as: “Why do young people cyberbully others?” and “What is the impact of cyberbullying on the perpetrator?”. Four members sat at each table and recorded their ideas on the flipchart paper. After five minutes they were invited to rotate to the next table to consider the next question. The flipchart sheets were then displayed and discussed by the group.

1. Gowran S. *Counted Out: Challenging Poverty and Social Exclusion.* Dublin: CDVEC Curriculum Development Unit and Combat Poverty Agency; 2002.

2. National Women's Council of Ireland. *Through the Looking Glass: A Guide to Empowering Young People to Become Advocates for Gender Equality.* Dublin: National Women's Council of Ireland; 2014.

3. Mc Menamin R, Tierney E, Mac Farlane A. Addressing the long-term impacts of aphasia: how far does the Conversation Partner Programme go? *Aphasiology.* 2015;29(8):889-913.

4. Chambers R. *Participatory Workshops: A sourcebook of 21 sets of ideas and activities.* UK: Earthscan; 2002.
